# Local Breast Cancer Spatial Patterning: A Tool for Community Health Resource Allocation to Address Local Disparities in Breast Cancer Mortality

**DOI:** 10.1371/journal.pone.0045238

**Published:** 2012-09-28

**Authors:** Dana M. Brantley-Sieders, Kang-Hsien Fan, Sandra L. Deming-Halverson, Yu Shyr, Rebecca S. Cook

**Affiliations:** 1 Department of Medicine, Vanderbilt University School of Medicine, Nashville, Tennessee, United States of America; 2 Department of Biostatistics, Vanderbilt University School of Medicine, Nashville, Tennessee, United States of America; 3 Department of Cancer Biology, Vanderbilt University School of Medicine, Nashville, Tennessee, United States of America; 4 Vanderbilt-Ingram Comprehensive Cancer Center, Vanderbilt University School of Medicine, Nashville, Tennessee, United States of America; 5 Social & Scientific Systems, Inc., Durham, North Carolina, United States of America; Yale School of Public Health, United States of America

## Abstract

Despite available demographic data on the factors that contribute to breast cancer mortality in large population datasets, local patterns are often overlooked. Such local information could provide a valuable metric by which regional community health resources can be allocated to reduce breast cancer mortality. We used national and statewide datasets to assess geographical distribution of breast cancer mortality rates and known risk factors influencing breast cancer mortality in middle Tennessee. Each county in middle Tennessee, and each ZIP code within metropolitan Davidson County, was scored for risk factor prevalence and assigned quartile scores that were used as a metric to identify geographic areas of need. While breast cancer mortality often correlated with age and incidence, geographic areas were identified in which breast cancer mortality rates did not correlate with age and incidence, but correlated with additional risk factors, such as mammography screening and socioeconomic status. Geographical variability in specific risk factors was evident, demonstrating the utility of this approach to identify local areas of risk. This method revealed local patterns in breast cancer mortality that might otherwise be overlooked in a more broadly based analysis. Our data suggest that understanding the geographic distribution of breast cancer mortality, and the distribution of risk factors that contribute to breast cancer mortality, will not only identify communities with the greatest need of support, but will identify the types of resources that would provide the most benefit to reduce breast cancer mortality in the community.

## Introduction

Despite advances in breast cancer prevention, early detection, and treatment, not all segments of the population benefit equally from these gains. For example, patients lacking health insurance have higher breast cancer mortality rates than other populations [1], [2], [3]. African American women are more likely to be diagnosed with advanced Stage IV breast cancer and experience higher breast cancer mortality rates than women in other ethnic groups [4], [5], [6], [7], [8], [9]. Additional factors contributing to increased mortality include low mammography screening rates [10], [11], [12], and low socioeconomic status (SES, defined herein as a median household income of less than $50,000 per year and an education level at or below high school level [13], [14], [15], [16]). To reduce breast cancer mortality in all segments of the population, it is necessary to define the populations in greatest need of additional interventions and to characterize disparities in underlying risk factors within that population that contribute to increased mortality. Once this information is in place, specific resources can be targeted toward the modifiable risk and mortality factors in a community-specific fashion, thereby increasing the likelihood of a beneficial outcome for the population as a whole.

Large nationwide studies have established a strong correlation between risk factors and breast cancer mortality. These risk factors include ethnicity, income, health insurance coverage, education, obesity, and screening (Breast Cancer Facts and Figures 2009–2010, American Cancer Society, Atlanta, GA). Many of these risk factors are demographically inter-related. For example, lower income often correlates directly with lack of health insurance coverage. Importantly, many of these risk factors also track geographically, such that there are geographic areas with high poverty rates, decreased education levels, an aging population, or a large African American population. While recent efforts have focused on identifying patterns in U.S. breast cancer mortality, mapping mortality rates alongside risk factors in a local, geographic context, or how previously identified risk factors may relate to breast cancer mortality within a local area [17], [18], [19], [20], [21], has been underinvestigated. An analysis of local patterns would allow for an objective, unbiased view of where resources might be allocated to address the greatest disparities and also to identify the types of resources that would likely benefit a specific region. Towards this aim, we analyzed demographic data from national and statewide datasets to examine the breast cancer mortality and risk factor patterns in the middle Tennessee area. These analyses were performed for counties within the middle Tennessee area, and subsequently for each ZIP code for the densely populated greater metropolitan Nashville area. Geographical areas of interest were identified as those with the endpoint of relatively high breast cancer mortality rates. Patterns were identified in specific geographical regions in which the breast cancer mortality rate exceeded what would be predicted from the breast cancer incidence rate, identifying potentially tractable targets for community resource allocation at the local level. We demonstrate through our study that analyses of known risk factors can be undertaken with existing data to map potential targets for intervention, which could serve as a model for community health resource allocation.

**Table 1:** Sociodemographic characteristic of the Middle Tennessee Community Profile Analysis.

County	% Low Income	Mean Income ($)	% College Graduates	% African American	% White	Total population
Cheatham	39.1	73,172	16.6	1.9	95.1	39,876
Davidson	41.2	83,993	22.8	27.1	65.9	635,710
Dickson	44.2	61,677	13.7	4.2	92.0	48,230
Maury	41.6	67,494	11.1	12.6	83.3	84,302
Montgomery	43.2	67,133	22.8	19.6	73.4	66,581
Robertson	33.0	65,440	12.8	8.6	89.6	259,048
Rutherford	37.2	74,622	26.1	11.4	81.8	158,759
Sumner	34.4	78,009	22.0	6.8	90.2	7,922
Trousdale	N.D.	N.D.	N.D.	N.D.	N.D.	N.D.
Williamson	18.0	132,946	50.2	4.9	90.4	176,838
Wilson	30.6	82,183	23.8	7.0	90.3	112,377
*Tennessee*	46.4	70,549	22.1	17.0	80.5	N.D.
*U.S.A.*	38.7	82,716	27.4	12.3	74.3	N.D.

Counties in the Middle Tennessee area used in the Middle Tennessee Community Profile Analysis (MTCPA) are listed in alphabetical order. Data regarding income, education, and racial demographics were compiled by the U.S. Census Bureau (2006–2008 American Community Survey) for all counties with a population greater than 60,000. Trousdale County was not a part of this dataset, and therefore the data points for Trousdale County were not determined (N.D.).

## Materials and Methods

### Hypothesis and Study Aims

The overall goal of this study was to determine if existing demographic data could be mined to uncover spatial patterns of breast cancer at the local level, which could then be applied to community health resource allocation. In order to address this hypothesis, we compared known breast cancer risk factors (listed below) with the endpoint of mortality across an 11-county area that encompasses urban, suburban, and rural communities, including the metropolitan Nashville area. Counties included Cheatham, Davidson, Dickson, Maury, Montgomery, Robertson, Rutherford, Sumner, Trousdale, Williamson, and Wilson. Each specific variable was examined for statistically significant correlations with mortality at both the county level and, within Metropolitan Nashville, at the ZIP code level.

**Table 2:** Breast cancer mortality risk factors in Middle Tennessee.

Quartile*	Menopausal population^1^	Breast Cancer Incidence^2^	Breast Cancer Mortality^3^	Stage IV Diagnoses^4^	Screening^5^	Uninsuredwomen^6^	Income^7^	Higher Education^8^	Racial Minority^9^
1	23.9%–29.5%	84.9–93.48	19.11–22.10	4.20%–4.29%	31.4%–36.6%	6.10%–11.70%	52,996–86,620	81.58%–90.10%	4.3%–9.4%
2	29.6%–30.8%	93.49–97.75	22.11–25.55	4.30%–4.44%	36.7%–37.8%	11.71%–15.15%	49,758–52,995	78.81%–81.57%	9.5%–12.0%
3	30.9%–31.3%	97.76–99.75	25.56–27.27	4.45%–4.52%	37.9%–39.4%	15.16%–15.95%	48,235–49,757	74.26%–78.80%	12.1%–18.4%
4	31.4%–34.0%	99.76–103.75	27.28–34.42	4.53%–5.00%	39.5%–42.1%	15.96%–18.30%	37,420–48,234	61.40%–74.25%	18.5%–37.9%

The population of Middle Tennessee was assessed using publically available data collected in 2009 describing demographic and breast cancer-related characteristics of the population.

* The value for each breast cancer risk factor was determined for each Middle Tennessee County, and counties were then ranked in numerical order from lowest to highest. The numerically ranked counties were then subdivided into quartiles, such that the three counties with the lowest risk factor values were placed in Quartile 1, and those with the highest were placed in Quartile 4. The range of risk factor values encompassed by each quartile are shown.

1 The percentage of the total female population in the county that is over the age of 50 years (a surrogate for menopause).

2 The breast cancer incidence per 100,000 women. ^3^Breast cancer mortality per 100,000 women.

4 The percentage of all breast cancers that were diagnosed at Stage IV.

5 The percentage of all breast cancers that were diagnosed without a prior mammographic screening.

6 The percentage of the female population lacking any form of health insurance.

7 The median household income.

8 The percentage of the population possessing higher than a high school level education.

9 The percentage of the population that is not Caucasian.

### Population Data

Demographic data from free, publically available national [Thomson Reuters, United States Census Bureau (2009)] and statewide [Tennessee Department of Health (2003–2007); Tennessee Cancer Coalition State Tumor Board Registry (2009)] datasets were queried for breast cancer mortality, incidence, age of female populations, number of Stage IV diagnoses, mammographic non-screening rates, females covered by insurance health insurance, median household income, education level, and ethnicity of the female population. These values were clustered for each county in Middle Tennessee and used to generate a community breast cancer profile for Middle Tennessee area. The variables assessed were selected based on known associations with breast cancer risk. The geographic boundary information was gained from the United States Census Bureau Maps and Cartographic Resources (2000), which are also publically available and free. The data were analyzed anonymously and not linked to any patient identifying information, thus alleviating privacy concerns.

**Table 3:** Counties rankings for each risk factor associated with breast cancer generated an integrated quartile score.

County Name	Scoring of breast cancer mortality and associated risk factors in middle Tennessee counties									
	Menopausal population	*Breast Cancer Incidence	*Breast Cancer Mortality	Stage IV Diagnoses	Screening	Uninsured women	Income	HigherEducation	Racial Minority	Integrated Quartile Score
Cheatham	2	2	4	1	2	1	1	3	1	23
Davidson	2	2	4	4	2	4	3	2	4	33
Dickson	3	1	3	2	4	3	4	4	1	29
Maury	3	4	2	4	3	4	2	3	3	34
Montgomery	1	2	2	4	3	2	2	1	4	25
Robertson	1	3	1	3	3	2	2	3	3	25
Rutherford	1	1	1	3	2	3	2	1	4	20
Sumner	4	3	2	2	1	2	2	2	2	25
Trousdale	4	4	4	4	4	4	4	4	2	42
Williamson	4	4	1	2	1	1	1	1	3	23
Wilson	2	3	3	2	1	1	1	2	2	23

Counties were ranked for each risk factor associated with breast cancer in numerical order according to data presented in Table 2 and Table 3. Based on their numerical ranking in each dataset category, each county was assigned a risk factor quartile score, with 1 indicating the lowest quartile, and 4 indicating the highest quartile. The quartile score for breast cancer mortality rate and breast cancer incidence rate was weighted double. The sum of the quartile scores of each category was caluclated for each county to generate the integrated quartile score. A high integrated quartile score is intended to reflect the county with the greatest need of breast cancer-related resources aimed at reducing breast cancer mortality.

**Table 4:** Correlation between incidence rate and post-menopausal proportion in Middle Tennessee Community.

County	Pearson Correlation	95% CI	p-value
Davidson	0.88	0.76∼0.94	<0.001
Cheatham	0.99	0.81∼1.00	0.002
Montgomery	1	0.99∼1.00	<0.001
Maury	0.95	0.68∼0.99	0.001
Dickson	0.94	0.56∼0.99	0.005
Wilson	0.79	−0.71∼1.00	0.210
Sumner	0.99	0.97∼1.00	<0.001
Rutherford	0.99	0.96∼1.00	<0.001
Robertson	0.58	−0.62∼0.97	0.302
Williamson	0.99	0.95∼1.00	<0.001

**Figure 1:**
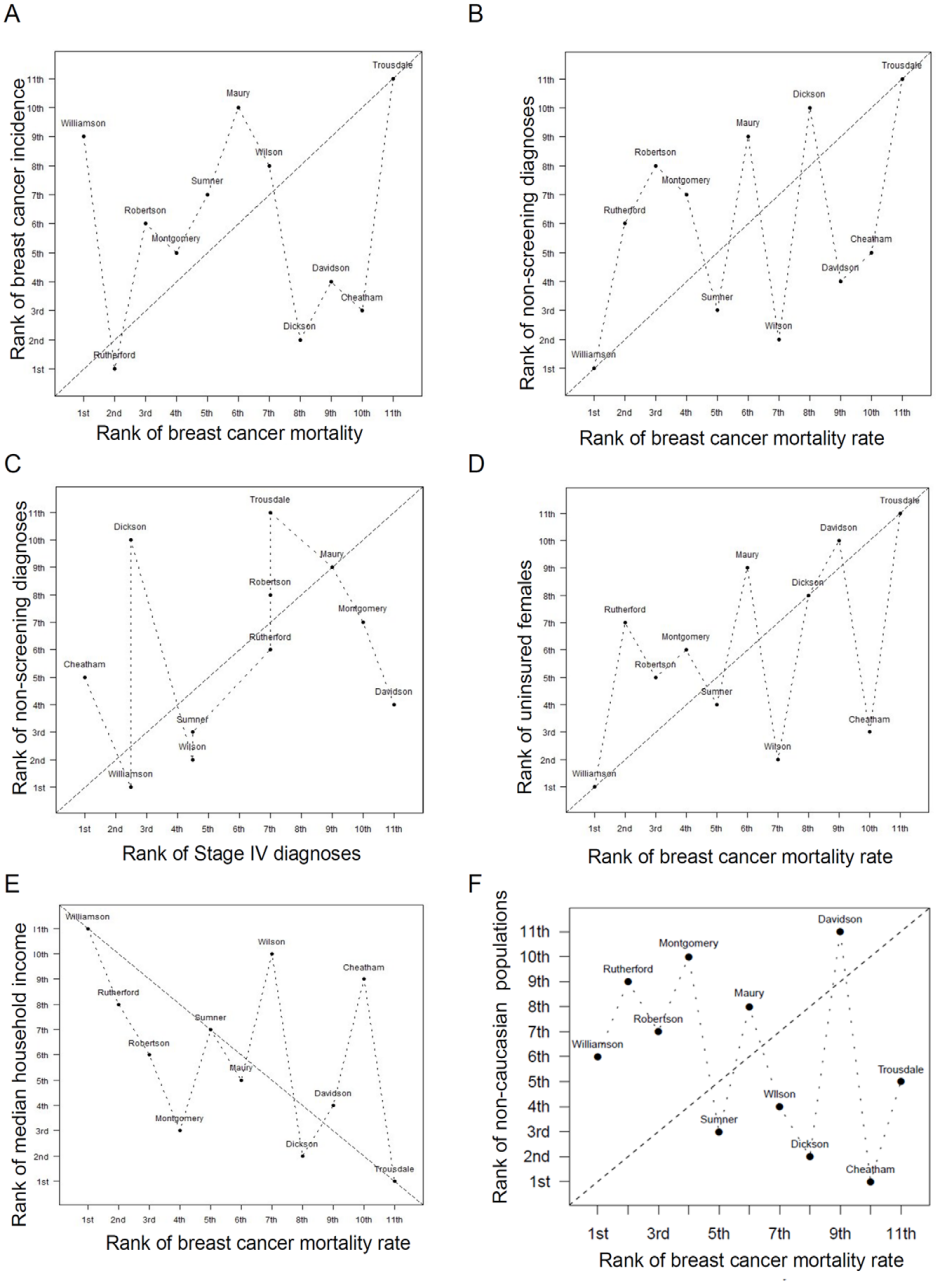
Breast cancer mortality rates in Middle Tennessee counties do not always correlate with age or breast cancer incidence. **A.** Correlation between breast cancer incidence rates and breast cancer mortality rates in 11 counties of Middle Tennessee. Spearman correlation is 0.06 with p-value equal 0.86. **B.** Correlation between breast cancer mortality rate and the percentage of breast cancers diagnosed without a prior mammographic screening. Spearman correlation is 0.35 with p-value equal 0.29. **C.** Correlation between percentage of breast cancers diagnosed at Stage IV patients and the percentage of breast cancers diagnosed without a prior mammographic screening in Middle Tennessee Counties. Spearman correlation is 0.26 with p-value equal 0.43. **D.** Correlation between breast cancer mortality rate and the percentage of the female population lacking health insurance in Middle Tennessee counties. Spearman correlation is 0.46 with p-value equal 0.15. **E.** Correlation between breast cancer mortality rate and median household income of Middle Tennessee counties. Spearman correlation is −0.46 with p-value equal 0.15. **F.** Correlation between breast cancer mortality rate and the percentage of the population that is non-white for each county in Middle Tennessee.

### Statistical Analyses

To assess potential correlations between breast cancer mortality with other risk factors within each middle Tennessee county or within the entire Middle Tennessee affiliate, population data and risk factors were clustered according to the ZIP codes geographically occupying each county. Correlations were examined by Pearson correlation test. Spearman’s tests were applied for examining the correlation between ranking of risk factors in individual counties. The risk factors were ranked in ascending order, from the county exhibiting the lowest risk factor measurement to the county exhibiting the highest risk factor measurement. All graphics and analyses were performed by R version 2.11.1. [R Development Core Team (2010). R: A language and environment for statistical computing. R Foundation for Statistical Computing, Vienna, Austria. ISBN 3-900051-07-0, URL http://www.R-project.org.].

**Table 5:** Correlation between incidence rate and mortality rate (A), incidence rate and percentage of patients with Stage IV breast cancer (B), and percentage of the female population that is non-white and breast cancer mortality (C) in Middle Tennessee Counties.

County	A. Incidence vs. Mortality			B. Stage IV diagnosis vs. Incidence			C. % Racial Minority vs. Mortality		
	Pearson Correlation	95% CI	p-value	Pearson Correlation	95% CI	p-value	Pearson Correlation	95% CI	p-value
Davidson	0.91	0.82∼0.96	<0.001	0.23	−0.15∼0.55	0.233	−0.04	−0.4∼0.33	0.233
Cheatham	1	0.97∼1.00	<0.001	0.93	0.25∼1.00	0.023	0.64	−0.56∼0.91	0.023
Montgomery	0.99	0.95∼1.00	<0.001	−0.70	−0.94∼0.01	0.054	−0.83	−.097∼−0.29	0.054
Maury	0.95	0.71∼0.99	0.001	0.71	−0.10∼0.95	0.075	0.25	−.062∼0.84	0.075
Dickson	0.96	0.65∼1.00	0.003	−0.06	−0.83∼0.79	0.903	0.12	−0.77∼.85	0.903
Wilson	0.96	−0.06∼1.00	0.044	0.35	−0.92∼0.98	0.648	0.14	−0.95∼0.97	0.648
Sumner	0.94	0.72∼0.99	<0.001	0.71	0.00∼0.94	0.050	0.65	−0.09∼0.93	0.050
Rutherford	0.99	0.95∼1.00	<0.001	−0.63	−0.88∼0.08	0.030	−0.72	−0.91∼−0.24	0.030
Robertson	0.73	−0.42∼0.98	0.159	0.97	0.64∼1.00	0.005	0.96	0.48∼1.00	0.005
Williamson	0.97	0.89∼0.99	<0.001	0.37	−0.34∼0.81	0.298	−0.47	−0.85∼0.23	0.298

Each variable was assessed for each ZIP code within each county. Based on The Pearson's Correlation between the indicated variables was calculated using datapoints obtained for each ZIP code within each county.

## Results

The Middle Tennessee region defined within this community profile analysis is an 11-county area that encompasses urban, suburban, and rural communities, including the metropolitan Nashville area. Census data, summarized in Table 1, indicate that socio-demographics within middle Tennessee vary widely amongst these eleven counties in terms of education level, mean household income, and the percentage of the population that is African American or white. Because a primary focus of this study was to define a method with which to identify communities that will benefit from additional resources, we examined breast cancer incidence rates for each county, ranking each county into quartiles based on the breast cancer incidence per 100,000 women. The counties with the lowest incidence rates were placed into Quartile 1, and those three counties with the highest incidence rates were placed in Quartile 4. We similarly assessed breast cancer mortality rates per 100,000 women in each county and the percentage of the female population that is post-menopausal, using the age of 50 years as a surrogate marker for menopause (Table 2). We ranked each county into quartiles ranging from lowest (Quartile 1) to highest (Quartile 4) in terms of breast cancer mortality rates and the percentage of the population that is menopausal (Table 3). For each county, there was a positive correlation between breast cancer incidence and the percentage of the female population that was menopausal (Table 4), although two of the eleven counties (Wilson and Robertson) did not show statistical significance of this correlation. These data suggest that breast cancer incidence can be higher in specific geographical areas, due in part to differences in the age of the female population in a geographic location, but perhaps due to other contributing factors as well.

**Table 6:** Geographical grouping (by ZIP code) of households within Davidson County according to median household income.

ZIP code	Percentage of Metro population	Median annualhousehold income
37228	26.4%	$13,523
37208	33.7%	$21,590
37203	35.3%	$24,663
37210	26.0%	$27,139
37240	0.0%	$30,000
37201	17.6%	$33,125
37207	20.0%	$33,259
37219	10.4%	$34,718
37206	25.5%	$34,967
37115	11.0%	$36,313
37212	8.7%	$38,968
37216	10.3%	$41,031
37209	10.8%	$42,030
37218	12.4%	$43,644
37217	7.6%	$43,713
37211	9.0%	$44,103
37204	6.4%	$48,895
37214	5.5%	$49,459
37189	6.1%	$50,922
37013	4.5%	$52,133
37080	6.5%	$52,199
37072	8.1%	$52,965
37076	5.1%	$53,983
37213	0.0%	$56,250
37138	4.9%	$60,938
37221	2.7%	$67,750
37205	2.6%	$74,067
37220	1.3%	$79,485
37215	2.1%	$82,635

Households within Davidson County (the greater metropolitan Nashville area) were grouped geographically according to ZIP code. The percentage of the metropolitan population residing within each ZIP code, and the median annual household income for each ZIP code was calculated based on data compiled by the U.S. Census Bureau (2006–2008 American Community Survey) for all ZIP codes within Davidson County.

**Table 7:** Integrated ranking (by ZIP code) of Davidson county subpopulations based on risk factors associated with breast cancer.

ZIP Code	Scoring of breast cancer mortality and associated risk factors in Davidson County ZIP codes							
	Breast cancer mortality rate*	Median house-hold income	Non-white population	Breast Cancer Diagnoses atStage IV	Population over 50 years of age	Incidence Rate*	Uninsured femalepopulation	Integrated Quartile Score
**37080**	3	2	2	2	4	3	3	25
**37189**	4	2	4	4	4	4	3	33
**37218**	4	3	4	4	4	4	4	35
**37209**	3	4	4	4	2	1	4	27
**37205**	4	1	2	2	4	4	2	27
**37221**	3	1	3	2	3	3	1	22
**37215**	4	1	1	2	4	4	1	25
**37204**	4	2	4	4	4	4	3	33
**37220**	4	1	1	1	4	4	1	24
**37211**	2	2	4	3	2	2	3	22
**37217**	2	3	4	4	1	1	3	21
**37210**	3	4	4	4	2	2	4	28
**37214**	4	2	3	3	3	3	2	27
**37076**	2	1	3	3	2	3	2	21
**37206**	3	4	4	4	2	2	4	28
**37216**	4	4	4	4	4	4	4	36
**37115**	4	4	4	4	3	3	4	33
**37207**	3	4	4	4	2	1	4	26
**37208**	4	4	4	4	2	1	4	28
**37225**	4	4	4	4	4	4	4	36
**37212**	3	4	3	4	1	1	4	24
**37203**	4	4	4	4	2	2	4	30
**37228**	4	4	4	4	4	4	4	36

Davidson county ZIP codes were ranked for each risk factor in numerical order according to: breast cancer incidence per 100,000 women, percentage of the female population over the age of 50 years, breast cancer mortality rate per 100,000 women, rate of Stage IV diagnosis, annual median income per household, the percentage of the female population lacking health insurance, and the percentage of the non-white population. Based on their numerical ranking in each dataset category, each ZIP code was assigned a risk factor quartile score, with 1 indicating the lowest quartile, and 4 indicating the highest quartile for each risk factor. The quartile score for breast cancer mortality rate was weighted double. The sum of the quartile scores of each category was calculated for each ZIP code to generate the integrated quartile score. A high integrated quartile score is intended to identify ZIP codes with the greatest need of breast cancer-related resources aimed at reducing breast cancer mortality.

We tested the hypothesis that breast cancer mortality rates would also vary widely between these eleven counties in middle Tennessee. We found that breast cancer mortality rates were not uniform throughout middle Tennessee (Tables 2, 3, and 4). This approach identified counties with high (Davidson, Cheatham, and Trousdale) and low (Williamson and Rutheford) breast cancer mortality rates. Comparing breast cancer mortality rates to incidence rates, it was clear that mortality did not correlate with incidence among Middle Tennessee counties (Fig. 1A, Spearman's ρ = 0.06 with P-value = 0.86). However, using ZIP code-based analysis of breast cancer mortality and incidence rates within individual counties, we identified counties in which positive correlations between breast cancer incidence and mortality were identified (*e.g*., Cheatham) or were not identified (*e.g*., Robertson, Table 5). These data suggest that other factors, some of which might be modifiable targets for community health resources, may contribute to higher mortality rates.

### Geographical Distribution of Late-stage Diagnosis and Breast Cancer Screening Patterns in Middle Tennessee

We assessed the rates of breast cancers diagnosed in women without previous mammographic screening, identifying Middle Tennessee counties with the highest (*e.g.*, Trousdale) and lowest (*e.g.*, Williamson) rates of breast cancers diagnosed in unscreened women. Importantly, a modestly positive correlation existed between breast cancer mortality rates and diagnoses occurring in unscreened women (σ = 0.33, P = 0.002, Fig. 1B), but exhibited geographical variation. It might be predicted that breast cancers diagnosed without prior mammographic screening would be higher in pre-menopausal populations, as pre-menopausal women are not routinely screened for breast cancer. While this pattern was often seen, it should be noted that one of the two counties exhibiting the highest rate of breast cancers diagnosed in the absence of mammographic screening also housed the highest proportion of post-menopausal women in Middle Tennessee. Therefore, the non-screening rate reported herein does not necessarily reflect a breast cancer population that is not routinely screened, but demonstrates a need for increased mammography screening in specific geographical areas, particularly rural areas.

The rate of Stage IV breast cancer diagnoses (number of Stage IV diagnoses per total number of breast cancer diagnoses) during 2009 for each of the 11 counties in the Middle Tennessee area (Tables 2 and 3) demonstrated wide geographic variability, identifying three counties in which Stage IV breast cancer diagnoses were the greatest. Interestingly, two of these three counties were among the Middle Tennessee counties with relatively lower breast cancer incidence rates, suggesting that in these counties, the rates of Stage IV diagnoses may exceed what would be expected based on the average rate of Stage IV diagnoses within a breast cancer patient population. Using geographical ZIP code boundaries to cluster breast cancer population data within each county, we determined that only 3 of the 11 counties in Middle Tennessee displayed the expected correlation between breast cancer incidence rates and Stage IV breast cancer diagnosis rates (Table 5). Therefore, counties exhibiting similar levels of Stage IV disease at the time of breast cancer diagnosis may have unique community characteristics underlying the failure to diagnose breast cancers earlier, such as utilization of mammographic screening. The rate of Stage IV breast cancer did not correlate with the rate of breast cancers diagnosed in unscreened women across all counties in middle Tennessee (Fig. 1C), although individual counties demonstrated a correlation among ZIP codes of their own county (*e.g.*, Williamson), suggesting that additional community characteristics must be considered.

Lack of health insurance coverage is a known breast cancer mortality risk factor [27], [28], [29], [30], [31]. We examined the geographic distribution of women lacking health insurance across Middle Tennessee counties (Table 2 and Table 3), finding that 6.10%–16.30% of the women in each county were without any health care coverage, including Medicaid or Medicare. Importantly, those counties with the largest presence of uninsured females also exhibited the highest breast cancer mortality rates (1D). Age can also be a confounding factor for this variable, as younger women are less likely to be uninsured and conversely, older women are more likely to have health insurance [32], [33]. This pattern was found to be true for some counties (Williamson and Sumner) but was not true for all counties. For example, the highest proportion of uninsured females was seen in Trousdale and Dickson Counties, which harbor the high proportions of post-menopausal females (Table 3). These data reveal geographic areas in the population wherein an aging, underinsured population might greatly benefit from resources that would make breast cancer detection and treatment financially feasible.

### The Impact of Socioeconomic Status (SES) on Breast Cancer Mortality in Middle Tennessee

We examined the sociodemographics that represent these geographically-distributed populations, identifying a wide variability in the geographic distribution between counties of median household income (Table 2). Socioeconomic status (SES), defined herein as a median household income of at least $50,000 per year and an education level above high school level (13–16), was used as a variable. A general trend identified a negative correlation between SES and breast cancer mortality (Fig. 1E). Lower income and education levels (*e.g.* Dickson and Trousdale) correlated with increased breast cancer mortality rates (Fig. 1E). However, not all counties demonstrated an inverse correlation between SES and breast cancer mortality, as high breast cancer mortality rates were seen in Cheatham County, which is in the highest SES quartile defined within this study.

### The Impact of Race/ethnicity on Breast Cancer Mortality in Middle Tennessee

Breast cancer mortality rates are often higher in women of African American descent compared to other ethnicities [4], [5], [6], [7], [8], [9]. Studies point to both genetic and socioeconomic factors underlying this observation [34], [35]. We investigated the geographic distribution of minority women (all non-white) for the entire middle Tennessee area (Table 2), and compared this to rates of breast cancer mortality. Upon examination of the counties in the entire middle Tennessee area, the Pearson’s correlation between racial minorities and breast cancer mortality was 0.105 (95% CI = −0.096 to 0.303; P = 0.297), while the Spearman correlation between racial minorities and breast cancer mortality of these 11 counties was −0.364 (P = 0.273; Fig. 1F), revealing no correlation between breast cancer mortality and racial minorities for the Middle Tennessee area. However, upon examination of individual counties within Middle Tennessee, we identified a significant positive correlation between the racial minority population and breast cancer mortality in 1/11 counties (Table 5), and a significant negative correlation between breast cancer mortality and the racial minority population in 2/11 counties.

### Geographical Assessment of Overall Breast Cancer Risk for Counties in Middle Tennessee

While correlations were identified between mortality and individual risk factors, allocation of resources to local geographically-based health care and outreach facilities will require the integration of all of these risk factors with breast cancer mortality. Using the quartile ranking of each statistic examined, we assigned a risk value to each statistic, with 1 being in the lowest risk quartile, and 4 being in the highest risk quartile. Breast cancer incidence and mortality rates were included as factors, and were weighted two-fold. Therefore, we used this approach to calculate for each county a numerical quartile score of breast cancer mortality risk factors (Table 3), producing a geo-demographical map to guide the placement of interventions aimed at reducing breast cancer mortality across the diverse communities of Middle Tennessee.

### High Resolution Geographical Analysis of Risk Factors in a Population-dense Metropolitan Area

A more detailed analysis of breast cancer and risk factor patterns within Davidson County was performed, based on the densely populated, metropolitan nature of Davidson County and the diverse populations and socioeconomic conditions that might exist within an urban environment (Table 6). We assessed population data representing each ZIP code within Davidson County, which revealed wide geographic variability in the rates of breast cancer incidence, the percentage of the female population over the age of 50 years, and breast cancer mortality. Examination of risk factors that contribute to increased breast cancer mortality indicated geographical diversity in the rate of stage IV breast cancer diagnosis, median household income, the percentage of the population lacking health care insurance, and the percentage of the population that was non-white (Table 6). Using the metric described above that assigns quartile scores for each of the risk factors contributing to breast cancer mortality, with incidence and mortality being weighted 2-fold, we were able to rank the geographic distribution of need within each geographic area delineated based on ZIP code (Table 7). This high-resolution analysis identified distinct geographical area with the highest integrated quartile risk factor score, indicating that these communities would benefit from additional resources aimed at breast cancer education, detection, and treatment.

One-to-one correlations between each risk factor and mortality in specific patients were not possible to analyze in these datasets. However given that this information is often unavailable for most geographically-defined populations, utilizing existing population-level data is an option to identify geographically distributed populations who disproportionately exhibit breast cancer mortality and the risk factors associated with increased breast cancer mortality. This analytical approach could be used to direct resources to these geographically-defined regions of need towards the goal of reduced breast cancer mortality. This approach could serve as a model for allocating limited resources, for addressing risk factor disparities that cause disparities in breast cancer mortality, and to produce a corresponding reduction in total breast cancer deaths.

## Discussion

Our analysis of breast cancer patterns in middle Tennessee revealed a significant overlap between age and breast cancer incidence rates. These data are consistent with the established correlation between breast cancer incidence and increasing age (Breast Cancer Facts and Figures 2009–2010, American Cancer Society, Atlanta, GA) and also provide an internal control for the validity of the patterns we observed in the Middle Tennessee area datasets. However, our approach revealed disparities in breast cancer mortality rates for urban (e.g. Davidson County), suburban (e.g. Williamson County) and rural (e.g. Cheatham County) areas that did not correlate directly with incidence and age. Therefore, we examined geographical distribution of late (Stage IV) breast cancer diagnosis rates in middle Tennessee. Early detection of breast cancer contributes substantially to the outcome of the patient, and has served to decrease breast cancer mortality rate over the last two decades [22], [23]. In contrast, breast cancers detected at stage IV often correlate with poor prognosis and decreased overall survival for patients with breast cancer [24], [25], [26]. We found disproportionate rates of late-stage (i.e., Stage IV) diagnoses for several counties, including Davidson, Maury, and Trousdale, which did not always correlate with incidence and age (e.g. Davidson County). We also examined the geographic distribution of health care access and socio-economic status (SES) because each of these factors is known to correlate with increased breast cancer mortality and to contribute to late diagnoses of breast cancer (Breast Cancer Facts and Figures 2009–2010, American Cancer Society, Atlanta, GA). Examination of geographic patterns of mammographic screening rates, levels of insurance coverage, indicators of SES, and ethnicity revealed that the basis for disparities in mortality and late stage diagnosis rates are geographically variable, suggesting interventions might be tailored towards the needs of each community.

For example, higher mortality rates in rural counties (e.g. Dickson, Maury, Trousdale) overlap with lower SES, including education, suggesting that educational resources coupled with greater access to care could benefit these populations. Access to information and educational materials may benefit specific population identified at the local level. Indeed, a recent study reported that Latinas exposed to cancer education were more likely to be up to date on Pap and mammography screening than community members who did not receive these materials [36]. Such services and information dissemination may be offered by patient navigators, defined by the NCI’s CRHD as providers of support and guidance offered to persons with abnormal cancer screening or a new cancer diagnosis in accessing the cancer care system; overcoming barriers; and facilitating timely, quality care provided in a culturally sensitive manner [http://grants.nih.gov/grants/guide/rfa-files/RFA-CA-05-019.html].

A common factor that overlapped with elevated mortality rates in urban Davidson County and rural Trousdale, Dickson, and Maury counties was a lack of medical insurance coverage, suggesting that this may be a critical barrier to breast cancer survival for several distinct populations in the Middle Tennessee area and an opportunity for intervention. Local resources, including public health departments, health care professionals, and local affiliates of national foundations (e.g. Susan G. Komen for the Cure, American Cancer Society, Avon Foundation, The National Breast Cancer Foundation, Breast Cancer Research Foundation), can provide resources in order to promote screening, increase breast cancer awareness in targeted communities (self-exam and disease risks, for example), and by making mammographic screening more readily available to an uninsured population.

Early detection relies on awareness of breast health, self-exams, and routine mammography. We therefore examined the non-screening rates in each of the eleven counties (Table 3). In spite of the recent controversy surrounding the benefit versus detrimental effects of mammography screenings, such as the potential for false positives, unnecessary biopsies, and overdiagnosis, ample evidence exists supporting the link between mammography screening and reduced breast cancer mortality rates [10], [11], [12]. Thus, programs that provide screening to under-served, at-risk patients identified through community profiling also have the potential to reduce mortality. Given reported associations between lower SES/lack of insurance and reduced mammography use in the U.S. [37], [38], [39], and the conversely higher SES/insurance coverage and increased mammography use [40], resources that increase access to and participation in mammography screening programs could benefit several overlapping target populations identified in middle Tennessee.

A critical barrier to successfully implementing such programs at the community level is identifying populations with the greatest need and/or the greatest risks, understanding where resources might have the greatest impact, and identifying which resources would be most effective. This is particularly important in the current economic environment in which donations and government services tend to diminish, leaving foundations and community health programs with more limited resources. Our community profile data suggest that the geographic distribution of breast cancer mortality in relation to known risk factors is a valuable tool for effective resource allocation. We demonstrate through our study that analyses of known risk factors can be undertaken with existing data to map potential targets for intervention, which could serve as a model for community health resource allocation. This model could serve to not only identifies communities with the greatest need of support, but also mortality-associated risk factor disparities within any given location community, thereby identifying modifiable targets that will provide the most benefit to the local area.
